# An unusual presentation of a patient with intrathoracic stomach: a case report

**DOI:** 10.4076/1757-1626-2-7514

**Published:** 2009-06-09

**Authors:** Yavuz M Bilgin, Hans E van der Wiel

**Affiliations:** Department of Internal Medicine, IJsselland HospitalCapelle aan den IJsselThe Netherlands

## Abstract

An intrathoracic stomach is the end stage of a hiatal hernial diaphragm and has a very low incidence. Frequently the diagnosis is made incidentally by endoscopic or radiographic investigations. There could be no clinical symptoms, however an intrathoracic stomach could be life treating. In this case we report a 61-year-old woman with an atypical presentation of an intrathoracic stomach. The patient had fever, night sweats and cough; the chest X-ray showed a retroperitoneal mass. A computed tomography scan was performed for determining the diagnosis of an intrathoracic stomach.

## Introduction

A hiatal hernia diaphragm is frequently found on radiographic or endoscopic investigations and usually needs no interventions. An intrathoracic stomach is the end stage of a hiatal hernia diaphragm and it is rarely found. An intrathoracic stomach could asymptomatic, although it could be associated with serious complications as incarceration, bleeding and perforation. Therefore a surgical intervention could be necessary. The purpose of this paper was to report a patient with an atypical presentation of an intrathoracic stomach.

## Case presentation

**Figure 1.**
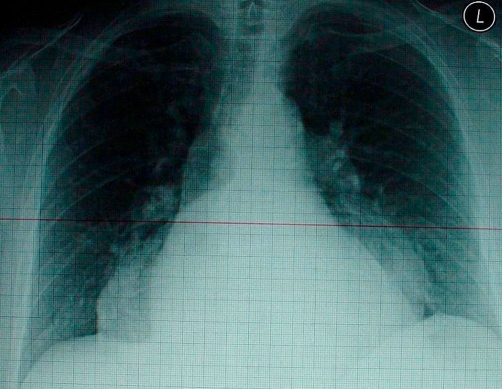
Posteroanterior chest X-ray of the patient showing a retroperitoneal large mass.

A 61-year-old Caucasian woman was referred to the emergency room of our hospital with fever, night sweats and non-productive cough since one week. She had weight lost of six kilograms in one month. Her medical history was unremarkable; she did not use medication and no recent travelling. Furthermore she did never smoked or drunk alcohol and had no gastro-intestinal symptoms. Physical examination showed an ill patient with a body temperature of 39.4°C, blood pressure of 110/70 mmHg and pulse of 100 beats/min. No lymph nodes were palpable, pulmonary and abdominal examination revealed no abnormalities. Relevant laboratory investigation results were: Hemoglobin 12.4 gm/dL, mean corpuscular volume 89 fl, leucocytes 7.7 × 10^9^ /l, with a normal differentiation, erythrocyte sedimentation rate 51 mm/hour, lactate dehydrogenase 173 U/l and C-reactive protein 184 mg/l. The posteroanterior chest X-ray showed no pulmonary infiltrations, but a large hypodense retroperitoneal mass was seen (Figure 1). Due to the condition of the patient no lateral chest X-ray could be performed.

**Figure 2.**
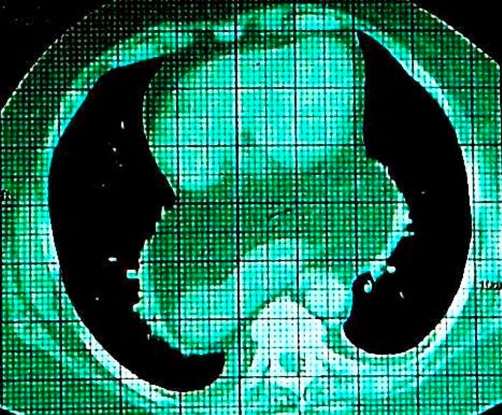
CT scan of the patient showing an intrathoracic stomach surrounded with fat. No other abnormalities were observed.

She was admitted to the department of Internal Medicine. At that time the patient was suspected with a mediastinal tumor, lymphoma or an ectopic thymoma, Therefore a computed tomography scan was performed to identify the mass. This revealed no pulmonary infiltrations, no enlarged lymph nodes or other abnormalities. However an intrathoracic stomach surrounded with fat was found (Figure 2). Our patient had a positive sputum culture with Klebsiella pneumoniae. She was diagnosed with respiratory tract infection and had been treated with antibiotics for 10 days. Her C-reactive protein normalized and she recovered well. She went home and is referred to the surgical outpatient clinic. The surgeon advised at this moment not to operate the intrathoracic stomach because she has no symptoms.

## Discussion

An intrathoracic stomach results from a hiatal hernia in which an important portion of the stomach had herniated through the diaphragm into the chest. The esopahgeal hiatal hernias are divided into 4 types [1,2]. The sliding hernia is the most commonly found type of esophageal hernia (represents 95% of all hiatal hernias) and is characterized by intrathroracic displacement of the gastro-esophageal junction (Type 1). Type 2 is the rolling or paraesophageal hernia, it shows displacement of the stomach fundus and anterior wall. Type 3 is a combination of types 1 and 2; the gastro-esophageal junction is displaced into the chest. Total herniation of the stomach represents the end stage of hiatal herniation and other organs could be herniated also to the chest (Type 4). The incidence of type 4 hiatal hernia compromises 0.3% of all hiatal hernias.

Most common clinical symptoms of an intrathoracic stomach are reflux and dysphagia. Due to food and air distension, patients with an intrathoracic stomach could present with postprandial pain, which can mimic angina and even myocardial infarction [3]. If the herniation is large compression of the lung may be the first symptom, which could lead to respiratory complications. Incarceration and strangulation of the intrathoracic stomach is a serious complication and could rarely lead to tension gastro-thorax [4]. Iron deficiency anemia could also be a presenting clinical symptom of an intrathoracic stomach. Anemia could be the result of mechanical irritation of the stomach leading to gastric erosions and ulcerations causing occult blood loss. Hematological and gastro-intestinal evaluations for other causes of blood loss are needed if iron deficiency anemia is present in patients with an intrathoracic stomach [5]. Our patient had no signs of blood loss in the laboratory investigations; therefore no further evaluations were performed. There is no indication for endoscopic investigation for the diagnosis of a hiatal hernia diaphragm. Most hiatal hernias are found incidentally on endoscopic or radiographic investigations. A fluid level on a chest X-ray suggests the presence of a hiatal hernia [1]. In our patient the presence of clinical symptoms with fever, cough, night sweats and weight loss and the absence of typical symptoms for an intrathoracic stomach had misled us. The differential diagnosis included mediastinal tumor, lymphoma or thymoma, however a computed tomography scan showed the final diagnosis of an intrathoracic stomach.

An asymptomatic intrathoracic stomach should be followed up. The treatment of a patient with a symptomatic intrathoracic stomach is a surgical intervention. Nowadays surgical repair is possible with laparoscopic techniques [6].

## Conclusion

Hiatal hernia diaphragms are commonly found, however an intrathoracic herniated stomach is rare. The diagnosis of an intrathoracic stomach should be considered in patients with a large mass on a chest X-ray. An intrathoracic stomach could be asymptomatic, however clinical symptoms could be life treating. If there are symptoms a surgical intervention is indicated.
